# Draft Genome Sequences of Three Strains of *Ehrlichia ruminantium*, a Tick-Borne Pathogen of Ruminants, Isolated from Zimbabwe, The Gambia, and Ghana

**DOI:** 10.1128/genomeA.00453-16

**Published:** 2016-06-16

**Authors:** Ryo Nakao, Frans Jongejan, Chihiro Sugimoto

**Affiliations:** a Unit of Risk Analysis and Management, Hokkaido University Research Center for Zoonosis Control, Sapporo, Hokkaido, Japan; b Laboratory of Parasitology, Department of Disease Control, Graduate School of Veterinary Medicine, Hokkaido University, Sapporo, Hokkaido, Japan; c Utrecht Centre for Tick-Borne Diseases (UCTD), FAO Reference Centre for Ticks and Tick-Borne Diseases, Faculty of Veterinary Medicine, Utrecht University, Utrecht, The Netherlands; d Department of Veterinary Tropical Diseases, Faculty of Veterinary Science, University of Pretoria, Onderstepoort, South Africa; e Division of Collaboration and Education, Hokkaido University Research Center for Zoonosis Control, Sapporo, Hokkaido, Japan; f Global Institution for Collaborative Research and Education, Hokkaido University, Sapporo, Hokkaido, Japan; g Department of Disease Control, School of Veterinary Medicine, University of Zambia, Lusaka, Zambia

## Abstract

The rickettsial bacterium *Ehrlichia ruminantium* is the causative pathogen of heartwater in ruminants. Here, we report the draft genome sequences of three strains of *E. ruminantium*, namely, the Crystal Springs strain from Zimbabwe, the Kerr Seringe strain from The Gambia, and the Sankat 430 strain from Ghana.

## GENOME ANNOUNCEMENT

Heartwater is a fatal disease of ruminants caused by an obligate intracellular bacterium *Ehrlichia ruminantium*. This rickettsial pathogen is transmitted by ticks of the genus *Amblyomma* (1) and is distributed in nearly all the countries of sub-Saharan Africa and on neighboring islands (2). The disease has also become established on some islands of the Caribbean, to which infected ticks were introduced through the livestock trade. Although a high level of genetic diversity was found among the strains in Africa by several genotyping methods (3, 4), only a limited number of strains have been sequenced so far (5, 6).

The following three *E. ruminantium* strains were sequenced in the present study: the Crystal Springs strain from Zimbabwe (7), the Kerr Seringe strain from The Gambia (8), and the Sankat 430 strain from Ghana (9). All strains were cultured in bovine aorta endothelial cells. When cell monolayers showed 70 to 90% lysis, the supernatant containing the free elementary bodies (EBs) was collected and centrifuged at 20,000 × *g* for 30 min. The pellet containing the EBs was then resuspended in phosphate-buffered saline, and the solution was filtered through a 5-µm membrane filter (Millipore, Bedford, MA, USA). The filtrate was treated with DNase I to remove any contaminating host-cell DNA. Bacterial DNA was then extracted using the NucleoSpin Tissue XS kit (Macherey-Nagel, Düren, Gemany) according to the manufacturer’s instructions.

The genomes were sequenced on the MiSeq platform (Illumina, San Diego, CA, USA) using a paired-end library with a 300-bp read length. After mapping the reads against each genome (phiX, *Bos taurus*, and *Mycoplasma* spp.) to remove contaminated data, *de novo* assembly was performed using Velvet version 1.2.10 (10) or CLC genomics workbench version 8.5.1 (Qiagen, Valencia, CA, USA). The assembled contigs were ordered to the Welgevonden (Erwo) strain using Mauve version 2.3.1 (11). Draft genome sequences of strains Crystal Springs, Kerr Seringe, and Sankat 430 comprised 34, 118, and 183 contigs (>500 bp), respectively (Table 1). The estimated genome sizes ranged from approximately 1,454 to 1,481 kb, and the *N*_50_ statistics ranged from 13,071 to 80,453 bp. The G+C content of each genome was calculated to be 27.5%.

**TABLE 1:** Summary of the draft genome sequences of three *Ehrlichia ruminantium* strains

Strain name	Accession no.	No. of contigs	Genome size (bp)	G+C content (%)	No. of CDSs^a^	No. of rRNAs	No. of tRNAs	Country of origin	Isolation year
Crystal Springs	BDDK01000001 to BDDK01000034	34	1,481,168	27.5	961	3	37	Zimbabwe	1990
Kerr Seringe	BDDL01000001 to BDDL01000118	118	1,453,658	27.5	997	3	36	The Gambia	2001
Sankat 430	BDDN01000001 to BDDN01000183	183	1,457,798	27.5	1,039	3	36	Ghana	1996

a CDSs, protein-coding sequences.

Prediction of protein-coding sequences (CDSs) and annotation were performed by the Microbial Genome Annotation Pipeline (http://www.migap.org). The number of CDSs varied between 961 and 1,039 (Table 1). All three strains possess a complete set of the major antigenic protein (*map1*) gene family, which has been associated with bacterial adaptation to mammalian hosts and vector ticks (12). The data presented here will facilitate comparative genomic analysis and expand our understanding of the genetic diversity of *E. ruminantium* circulating in the African continent, which is useful for the appropriate formulation of the vaccine against heartwater.

### Nucleotide sequence accession numbers.

The nucleotide sequence accession numbers for DDBJ/EMBL/GenBank are found in Table 1. The versions described in this paper are the first versions.

## References

[1] Walker JB, Olwage A 1987 The tick vectors of *Cowdria ruminantium* (Ixodoidea, Ixodidae, genus *Amblyomma*) and their distribution. Onderstepoort J Vet Res 54:353–379. 3329325

[2] Uilenberg G 1983 Heartwater (*Cowdria ruminantium* infection): current status. Adv Vet Sci Comp Med 27:427–480. 6359836

[3] Nakao R, Magona JW, Zhou L, Jongejan F, Sugimoto C 2011 Multi-locus sequence typing of *Ehrlichia ruminantium* strains from geographically diverse origins and collected in *Amblyomma variegatum* from Uganda. Parasit Vectors 4:137. doi:10.1186/1756-3305-4-137. 21762509 PMC3151223

[4] Nakao R, Morrison LJ, Zhou L, Magona JW, Jongejan F, Sugimoto C 2012 Development of multiple-locus variable-number tandem-repeat analysis for rapid genotyping of *Ehrlichia ruminantium* and its application to infected *Amblyomma variegatum* collected in heartwater endemic areas in Uganda. Parasitology 139:69–82. doi:10.1017/S003118201100165X. 22008706

[5] Collins NE, Liebenberg J, de Villiers EP, Brayton KA, Louw E, Pretorius A, Faber FE, van Heerden H, Josemans A, van Kleef M, Steyn HC, van Strijp MF, Zweygarth E, Jongejan F, Maillard JC, Berthier D, Botha M, Joubert F, Corton CH, Thomson NR, Allsopp MT, Allsopp BA 2005 The genome of the heartwater agent *Ehrlichia ruminantium* contains multiple tandem repeats of actively variable copy number. Proc Natl Acad Sci USA 102:838–843. doi:10.1073/pnas.0406633102. 15637156 PMC545511

[6] Frutos R, Viari A, Ferraz C, Morgat A, Eychenié S, Kandassamy Y, Chantal I, Bensaid A, Coissac E, Vachiery N, Demaille J, Martinez D 2006 Comparative genomic analysis of three strains of *Ehrlichia ruminantium* reveals an active process of genome size plasticity. J Bacteriol 188:2533–2542. doi:10.1128/JB.188.7.2533-2542.2006. 16547041 PMC1428390

[7] Byrom B, Yunker CE, Donovan PL, Smith GE 1991 In vitro isolation of *Cowdria ruminantium* from plasma of infected ruminants. Vet Microbiol 26:263–268. doi:10.1016/0378-1135(91)90019-C. 2024445

[8] Faburay B, Munstermann S, Geysen D, Bell-Sakyi L, Ceesay A, Bodaan C, Jongejan F 2005 Point seroprevalence survey of *Ehrlichia ruminantium* infection in small ruminants in The Gambia. Clin Diagn Lab Immunol 12:508–512. doi:10.1128/CDLI.12.4.508-512.2005. 15817758 PMC1074385

[9] Bell-Sakyi L, Koney EBM, Dogbey O, Abbam JA, Aning KG 1997 Isolation and *in vitro* cultivation in Ghana of *Cowdria ruminantium*, the causative agent of heartwater, p. 46–51. In Proceedings of the W.A.C.V.A/G.V.M.A. Conference, Ministry of Food and Agriculture, Accra, Ghana.

[10] Zerbino DR, Birney E 2008 Velvet: algorithms for de novo short read assembly using de Bruijn graphs. Genome Res 18:821–829. doi:10.1101/gr.074492.107. 18349386 PMC2336801

[11] Darling AE, Mau B, Perna NT 2010 progressiveMauve: multiple genome alignment with gene gain, loss and rearrangement. PLoS One 5:e11147. doi:10.1371/journal.pone.0011147. 20593022 PMC2892488

[12] Postigo M, Taoufik A, Bell-Sakyi L, Bekker CPJ, de Vries E, Morrison WI, Jongejan F 2008 Host cell-specific protein expression in vitro in *Ehrlichia ruminantium*. Vet Microbiol 128:136–147. doi:10.1016/j.vetmic.2007.09.023. 18006251

